# A Facile Fabrication of High Toughness Poly(lactic Acid) via Reactive Extrusion with Poly(butylene Succinate) and Ethylene-Methyl Acrylate-Glycidyl Methacrylate

**DOI:** 10.3390/polym10121401

**Published:** 2018-12-17

**Authors:** Bin Xue, Hezhi He, Zhiwen Zhu, Jiqian Li, Zhaoxia Huang, Guozhen Wang, Ming Chen, Zhiming Zhan

**Affiliations:** 1 National Engineering Research Center of Novel Equipment for Polymer Processing, School of Mechanical and Automotive Engineering, South China University of Technology, Guangzhou 510640, China; xbinscut@163.com (B.X.); mrobito@163.com (Z.Z.); ljqnx21@163.com (J.L.); gjoewang@163.com (G.W.); chenm243@163.com (M.C.); 201721002896@mail.scut.edu.cn (Z.Z.); 2 Key Laboratory of Polymer Processing Engineering, Ministry of Education, School of Mechanical and Automotive Engineering, South China University of Technology, Guangzhou 510640, China

**Keywords:** poly(lactic acid), poly(butylene succinate) (PBS), ethylene-methyl acrylate-glycidyl methacrylate, in situ reactive, compatibility, toughening mechanism

## Abstract

As is an excellent bio-based polymer material, poly(lactic acid) (PLA)’s brittle nature greatly restricts its extensive applications. Herein, poly(butylene succinate) (PBS) was introduced to toughening PLA by melt blending using a self-made triple screw extruder through in situ reactive with ethylene-methyl acrylate-glycidyl methacrylate (EGMA). The effect of EGMA concentrations on the mechanical properties, morphology, interfacial compatibility of PLA/PBS blends were studied. Fourier transform infrared (FT-IR) results demonstrated that the epoxy group of EGMA reacts with the hydroxyl groups of PLA and PBS, which proved the occurrence of interfacial reactions among the tri-component. The significantly improved compatibility between PLA and PBS after EGMA incorporation was made evident by scanning electron microscope (SEM) characterization results. Meanwhile, the contact angle test predicted that the EGMA was selectively localized at the interface between PLA and PBS, and the result was verified by morphological analysis of cryofracture and etched samples. The EGMA improves the compatibility of PLA/PBS blends, and consequently leads to a significantly increased toughness with the elongation at break occurring 83 times more when 10 wt % EGMA was introduced than neat PLA, while impact strength also enhanced by twentyfold. Ultimately, the toughening mechanism of PLA based polymers was established based on the above analysis, exploring a new way for the extensive application for degradable material.

## 1. Introduction

In recent years, tens of billion tons of plastics are widely used in industrial, agricultural, national defense, healthcare and other fields [1], where most are extracted from petroleum resources. With the increased usage of pertrochemical-based plastics, it causes serious environmental pollution and wastes of oil resources [2,3]. Therefore, the environmentally friendly biobased and biodegradable plastics as well as less persistent and toxic, low carbon green have attracted wide concerns [4,5].

In the market, there are many biodegradable plastics such as poly(butylene succinate) (PBS) [6], poly(butyleneadipate-co-terephthalate)(PBAT) [7], poly(hydroxy alkanoate) (PHA) [8], poly(lactic acid) (PLA), etc. Among these, PLA is the most promising bio-based plastic, due to its excellent biocompatibility and biodegradability. In addition, PLA can be obtained from corn, wheat, and cassava resources [9,10], and degraded by microorganisms to CO_2_ and H_2_O. Moreover, owing to the advantages of outstanding mechanical properties and heat resistance [11,12], PLA is an ideal green polymer material can be used in packaging, architecture and biomedical fields [13,14]. However, the brittle with low elongation at break and low notched impact strength restrict its extensive applications. In order to enhance the toughness of PLA, many scientists have adopted various methods such as chemical copolymerization, melt blending, and nano-filling [15]. Among them, blending with flexible material is the simplest and most effective method [3,12,14].

The flexible materials used for toughening, such as non-degradable plastics like acrylonitrile -butadiene-styrene(ABS) [16], linear low-density polyethylene (LLDPE) [17], ethylene-propylene-piene monomerb (EPDM) [18], and degradable plastics such as PBAT [7], poly(capro lactone)(PCL) [19], poly(butylenes-succinate- co-adipate) (PBSA) [20], etc., have been introduced to modify PLA with a different toughening-level. However, some of these materials do not indicate a significant toughening effect, due to incompatibility with PLA, which results in phase-separated morphologies between the bi-phase, and weakens the performance of the blend [14,20,21]. 

In order to fabricate high toughness composites, poly(butylene succinate) (PBS) is considered for blending with PLA, which is a highly flexibility biodegradable material with comprehensive properties are similar to acrylonitrile -butadiene-styrene(ABS) and polypropylene (PP). Previously, Raksit et al. [22] and Yokohara et al. [23] fabricated the PLA/PBS binary blends with considerable mechanical properties, but the poor compatibility between PLA and PBS induced phase-separated morphologies. Therefore, it is necessary take some measures to improve the compatibility of these two polymers. At present, an in situ reaction compatibilization technique is the most effective method for improving phase-separated morphologies of incompatible systems, which has been used by scholars. Ojijo et al. [20] used triphenyl phosphite in the reactive compatibilization of PLA and PBSA, the notched impact strength of the binary blend obviously improved from 6.76 kJ/m^2^ to 16.4 kJ/m^2^ with 2 wt % triphenyl phosphite, and the morphology of PLA/PBSA blends evolved from “island–sea” structure to a more uniform type. Liu et al. [24] added a small amount of Poly(D-lactide) (PDLA) into PLLA/TPU blends, and it can be observed that the in situ reactive among the two components with PDLA was also added. Meanwhile, the impact strength increased significantly from 9.6kJ/m^2^ to 63.2 kJ/m^2^ with 15 wt % TPU. The effect of Polyolefin elastomer (POE) on the properties of PLA/PA11 blends was researched by Heshmati al. [25] and discovered that the PEO can improve the interfacial compatibility remarkably, the impact strength of PLA/PA11 with 15 wt % PEO demonstrating 17.5 times and three times higher than neat PLA and PA11, while elongation at break improved from 6% of neat PLA to 275% of PLA/PA11 blend.

A series of studies have shown that the reactive compatibilization has a significant change in the phase morphology of the PLA based polymer, which improves the toughness [24,25,26,27]. However, so far, there has been little research on the reaction compatibilization of the PLA/PBS blend. Thus, it is necessary to carry out related research. In this paper, we focused on reactive blending of PLA/PBS blend with ethylene-methyl acrylate-glycidyl methacrylate (EGMA) by a self-made triple screw extruder, and highly-toughened PLA based ternary blends were fabricated. Our goal is to study the effect of EGMA on the morphology and mechanical properties of PLA/PBS blends, and analyze the relationship between tensile toughness, impact toughness and compatibility, phase morphology, establishing the toughening mechanism of PLA/PBS/EGMA ternary blends.

## 2. Materials and Methods 

### 2.1. Materials

Commercial PLA (4032D) was purchased from NatureWorks LLC (Blair, NE, USA) with a density of 1.24 g cm^−3^ and 1.2–1.6 wt % D-isomer lactide. PBS pellets (3001MD) were supplied by Show a High Polymer Co., Ltd. (Tokyo, Japan) with the density of 1.26 g cm^−3^. EGMA (LOTADER AX 8900) was provided from Arkema Investment Co., Ltd. (Shanghai, China) with the density of 0.944 g cm^−3^ and contained 24 weight fraction (wt) % methyl acrylate and 8 wt % glycidyl methacrylate. 

### 2.2. Fabrication of Samples

The PLA was dried at 80 °C in an oven for 10 hours, while PBS and EGMA pellets were dried at 50 °C in oven for 10 h. The PLA/PBS and PLA/PBS/EGMA blends with different weight fraction were fabricated by melt blending using a self-made triple screw extruder. The extrusion barrel of the extruder has 8 heating zones from hopper to die, the temperature profile was 170 °C, 180 °C, 190 °C, 190 °C, 190 °C, 190 °C, 190 °C and 180 °C, the speed of rotor was 80 rpm. Subsequently, the kneaded polymers obtained were compression molded into standard dumbbell-shaped specimens and rectangular specimens for tensile testing and impact testing at 195 °C and 15 MPa.

### 2.3. Mechanical Testing 

The tensile properties of each blend were tested by an Instron Universal Testing Machine (model 5566, Norwood, MA, USA), with crosshead speed of 20 mm/min according to GB/T1040-2006. The impact strength was tested by the PIT501B-Z pendulum impact tester (Zwick5117, Zwick GmbH, Ulmer, Germany) in line with GB/T 1843-2008. The average mechanical property values are obtained from more than five specimens.

### 2.4. Scanning Electron Microscopy (SEM)

The morphology of cryo-fractured surfaces, etched surfaces and impact-failure surfaces of each sample was observed by SEM (S-3700N, Hitachi, Tokyo, Japan). To obtain the morphology of cryo-fractured surfaces, the rectangular samples were fractured after 60 min soaking in liquid nitrogen, and then all surfaces were covered with gold, and subjected to SEM imaging at an applied voltage of 5 kV.

### 2.5. Fourier Transform Infrared (FT-IR) Spectroscopy 

The FT-IR spectroscopy (Nicolet Nexus 670, Dongguan, China) was used to study the molecular group reactions between PLA and PBS with EGMA. Take a small amount of PLA, PBS, EGMA, PLA/PBS, PLA/PBS/EGMA samples, and press the samples into films with a hot stamper. Scan each film of all samples by the FT-IR spectroscopy with 64 scans a range of 400 to 4000 cm^−1^.

### 2.6. Contact Angle Testing

Contact angle test was conducted to study the compatibility between the tri-component by a contact angle measurement system (OCA40Micro, Dataphysics, Stuttgart, Germany). In addition, 5 mL of distilled water (H_2_O) and diiodomethane (CH_2_I_2_) were dropped on the PLA, PBS, and EGMA samples. The static contact angle data are recorded by a OCA40Micro measurement system, respectively. The tested data of contact angle is the average of more than five different positions on each specimen.

## 3. Results and Discussion

### 3.1. The FT-IR Analysis

**Figure 1:**
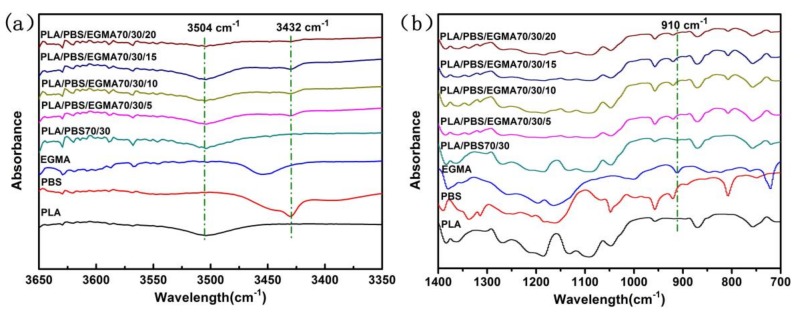
FT-IR spectra of specimens: (**a**) 3650–3350 cm^−1^; (**b**) 1400–700 cm^−1^.

In this work, a compatibilizer agent EGMA was added to PLA/PBS blends to improve compatibility of these two phases. During the melt compounding, EGMA contains epoxy groups that could react with the hydroxyl groups as discussed in the previous literature by Zhang et al. [28] and Worasak et al. [29]. To demonstrate that the in situ reaction exists in PLA/PBA/EGMA blends, the FT-IR analysis was employed as shown in Figure 1. Clear absorption peaks of hydroxyl groups of PLA and PBS at 3504 cm^−1^ and 3432 cm^−1^ can be seen in Figure 1a. With incorporation of 30 wt % PBS to PLA, the absorption peak still appears in a PLA/PBS blend. However, it is worth noting that the absorption peaks at 3504 cm^−1^ and 3432 cm^−1^ gradually decrease with the increase of EGMA content, which is even almost invisible with 20 wt % EGMA loading. Meanwhile, it can be observed that the absorption peak of epoxy groups of EGMA at 910 cm^−1^ was presented in Figure 1b, while the absorption peak disappeared in the PLA/PBS/EGMA blends, which proves that the epoxy group of EGMA reacts with the hydroxyl groups of PLA and PBS.

**Figure 2:**
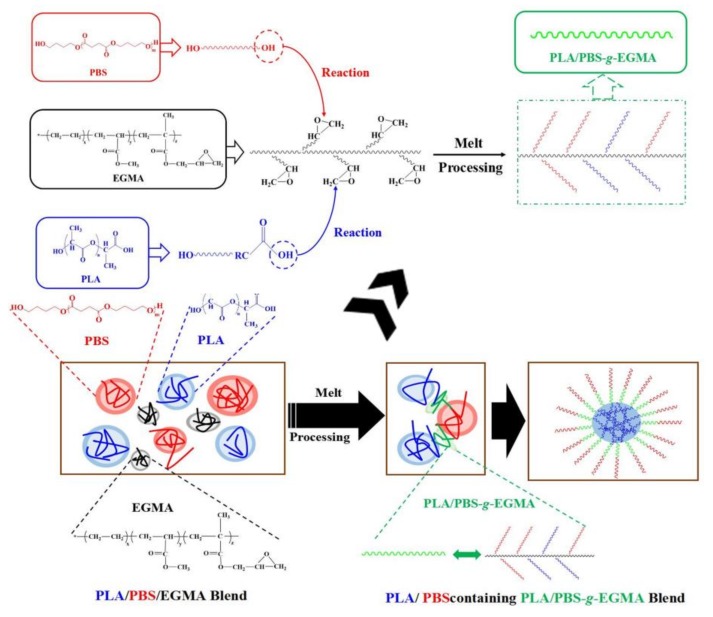
Schematic diagram of possible reaction routes in PLA/PBS blend with the inclusion of EGMA.

The schematic diagram of possible reaction routes of PLA/PBS blends with the inclusion of EGMA is illustrated in Figure 2. As discussed above, melt compounding promoted reactions of EGMA with PLA and PBS. As a result, PLA/PBS-g-EGMA co-polymers could be formed at the PLA-PBS interface and served as bridges connecting these phases. Thus, the poor interfacial adhesion between immiscible PLA and PBS phases may be significantly improved by the inclusion of EGMA. Moreover, the number of bridges increased with EGMA loading and thus led to an improved compatibility, which allowed forces acting during the impact to be transferred from the brittle PLA matrix to the ductile PBS phase. The above transfer led to an elongational deformation in the PBS phase and, consequently, to fracture energy dissipation. Whether this assumption is correct will be verified in the following discussion.

### 3.2. Mechanical Properties Analysis 

**Figure 3:**
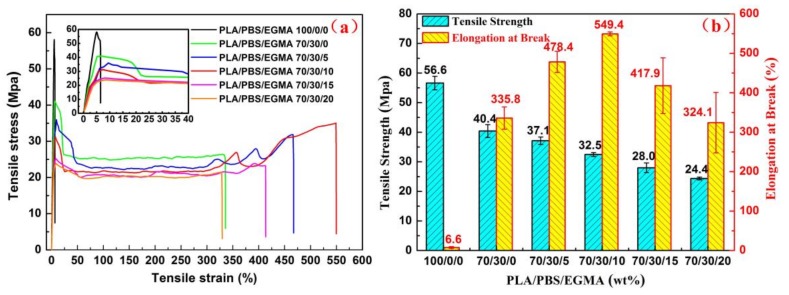
The tensile properties of neat PLA, PLA/PBS and PLA/PBS/EGMA blends: (**a**) strain–stress curves; (**b**) tensile strength and elongation at break.

The tensile properties of neat PLA, PLA/PBS and PLA/PBS/EGMA blends are shown in Figure 3. It can be seen that strain–stress curves (Figure 3a) and tensile strength and elongation at break (Figure 3b) indicate that the neat PLA is a brittle material, without obvious yield behavior and showing poor elongation at break (ε = 6.64%) with a high tensile strength (σ = 56.6 MPa). When incorporating 30 wt % PBS into PLA, the blend shows significant yielding with a brittle to ductile transition exhibiting increased elongation at break (*ε* = 335.8%) but decreased tensile strength (σ = 40.4 MPa), which indicates that the soft PBS added to PLA caused increased ductility but a decrease of stiffness. When the EGMA was added to a PLA/PBS blend, the tensile strengths are 37.1 MPa, 32.5 MPa, 28.0 MPa, and 24.4 MPa for samples with 5 wt %, 10 wt %, 15 wt % and 20 wt % EGMA, respectively. On the contrary, the elongation at break of blends has a remarkable increase from 478.4% for 5 wt % EGMA to a maximal value of 549.4% for 10 wt % EGMA and exhibits superior stretchability of 83 times higher than neat PLA, which represents enhanced ductility. With further increase of EGMA contents to 15 wt % and 20 wt %, the elongations at break slightly drop to 417.9% and 324.1%, respectively. 

**Figure 4:**
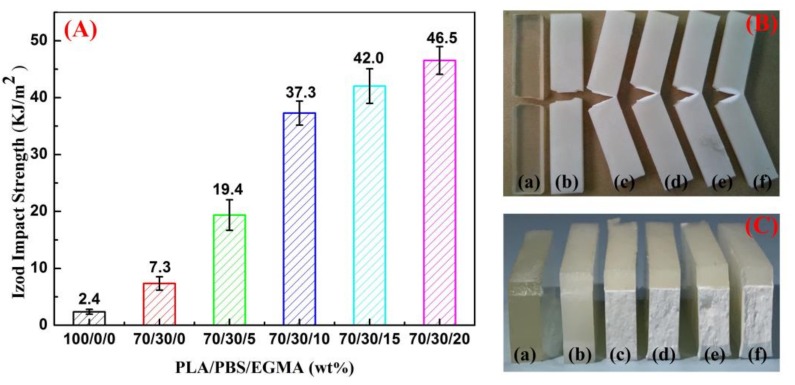
The impact properties of neat PLA, PLA/PBS and PLA/PBS/EGMA blends: (**A**) impact strength; (**B**–**C**) deformed impact samples: (**a**) neat PLA; (**b**) PLA/PBS 70/30; (**c**–**f**) PLA/PBS/EGMA (70/30/x): (**c**) 5 wt %; (**d**) 10 wt %; (**e**) 15 wt %; (**f**) 20 wt %.

**Figure 5:**
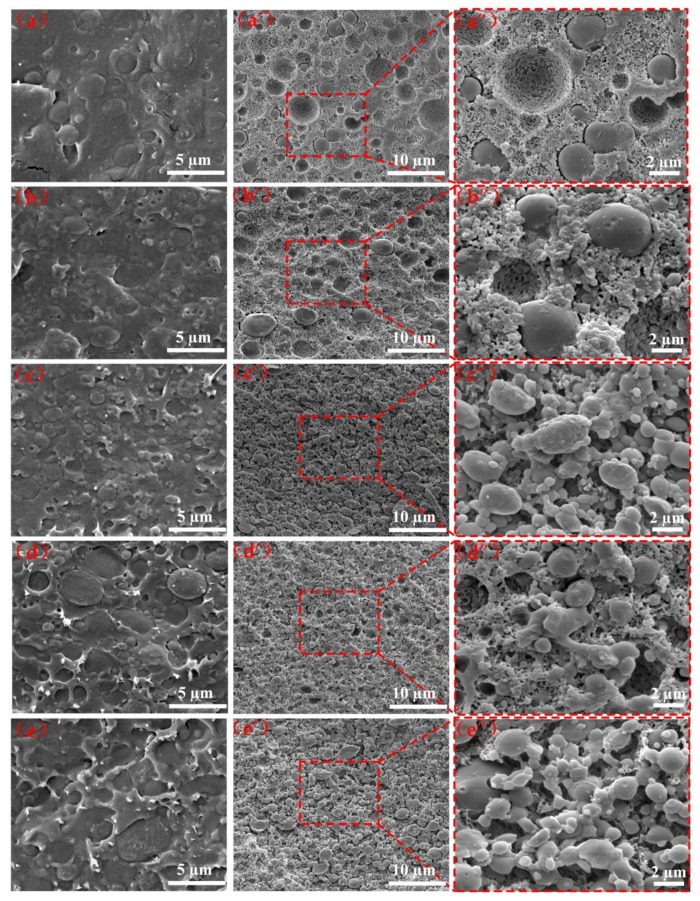
SEM images of cryofracture and PLA amorphous were etched cryofracture samples: (**a**–**e**) cryofracture surfaces; (**a’**–**e’,a’’**–**e’’**) etcyed cryofracture surfaces: (**a**–**a’’**) PLA/PBS 70/30; (**b**–**b’’**) PLA/PBS/EGMA 70/30/5; (**c**–**c’’**) PLA/PBS/EGMA 70/30/10; (**d**–**d’’**) PLA/PBS/EGMA 70/30/15; (**b**–**b’’**) PLA/PBS/EGMA 70/30/20.

It is well known that the impact property is an important evaluating indicator of a material’s toughness. The notched impact properties of neat PLA, PLA/PBS and PLA/PBS/EGMA blends are shown in Figure 4. The neat PLA exhibited a low impact strength of 2.4 kJ/m^2^ due to the brittle nature. With incorporation of 30 wt % PBS to a PLA matrix, the impact strength increased by three times with a value of 7.3 kJ/m^2^, owing to the fact that the elastic PBS can absorb more impact energies. However, this binary blend exhibited typical brittle behavior and featured few plastic deformations prior to failure, presenting SEM images of PLA/PBS impact fracture surface (Figure 5b), and leading to the insignificant increase since PLA and PBS phases were not very compatible. On the contrary, the impact strength obviously increases with incorporation of 5 wt % EGMA to 19.4 kJ/m^2^. The impact strength remarkably improved in particular, reaching 46.5 kJ/m^2^ with incorporation of 20 wt % EGMA approximately 20 times more than neat PLA, which presented the occurrence of a brittle-to-ductile transition.

### 3.3. Morphological Analysis 

It is known that the morphology, including particle size and distribution of the dispersed phase in the matrix, play an important role in the mechanical properties of polymers. In order to more deeply investigate the relationship between toughness mechanism and microstructure evolution of PLA/PBS/EGMA ternary blends, the cryo-fractured surfaces were etched by a sodium hydroxide–methanol−water solution in order to eliminate the PLA amorphous in the blends from being implemented [30]. The SEM images of cryofracture surfaces and PLA amorphous etched cryofracture surfaces of the PLA/PBS blend and the PLA/PBS/EGMA blends with different weight fraction EGMA are shown in Figure 5.

It can be observed from Figure 5a that the 70/30 sample shows a typical “sea–island” structure, where the PBS sphere particles were unevenly dispersed in the continuous PLA phase. In the etched cryo-fracture surfaces shown in Figure 5a’,a”, it is clear that large voids surrounding spherical PBS nodules with spherical PBS phases are surrounded by PLA crystals with no interconnection between PLA and PBS phases. Investigating the morphology further, it can be found that even more PBS particles detached from the PLA matrix, which proved that the two phases were immiscible, which can consequently result in low mechanical properties of the binary blend. With the incorporation of 5 wt % EGMA, the morphology shown in Figure 5b of the ternary blend has changed somewhat with interfaces becoming obscured between PLA and PBS phases. Although some of the PBS particles are still being pulled out, there are numerous interconnections between the PLA crystal and the PBS phase obtained that are presented in Figure 5b’,b”, which indicated that the EGMA improves the compatibility of the two phases. With the further addition of EGMA from 10 wt % to 20 wt % into the PLA/PBS blend, the two-phase interface has gradually disappeared, as shown in Figure 5c–e, and there are many white substances around the PBS particles, indicating that the EGMA takes part in the reactions with PLA and PBS, and serving as a bridge to connect the two, which improves the phases interfacial adhesion. Meanwhile, it is clearly observed that the dispersed PBS phase transforms from the spherical shape to a shish-like shape with higher EGMA added to the matrix shows PLA amorphous etched cryofracture surfaces (Figure 5c”–e”). The spherical PBS and PLA crystals are connected by EGMA, which strongly proves that polymer compatibility is improved.

**Figure 6:**
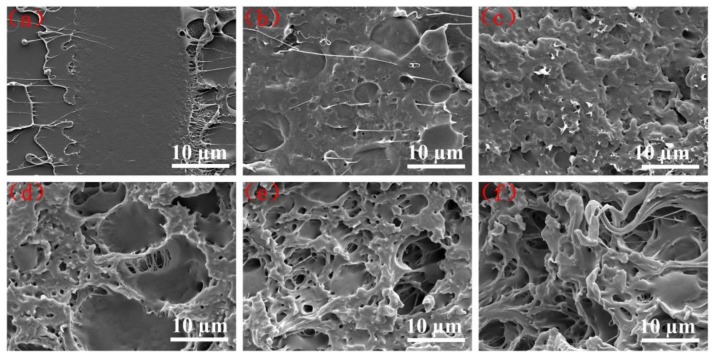
SEM micrographs of impact fracture surfaces: (**a**) neat PLA; (**b**) 70/30; (**c**) 70/30/5; (**d**) 70/30/10; (**e**) 70/30/15; (**f**) 70/30/20.

The impact toughness of PLA increased significantly with the addition of PBS and EGMA as discussed above, and the microimages of impact failure surface are indicated in Figure 6. In Figure 6a, the neat PLA exhibited smooth surfaces similar to the cryofracture surface, indicating typical brittle behavior. With the addition of PBS, the impact surface showed little plastic deformation presented in Figure 6b, as evidence of an insignificant toughening effect caused by the poor interfacial adhesion between PLA and PBS. When 5 wt % EGMA was added into a PLA/PBS blend, several plastic deformation areas were observed in Figure 6c, indicating an improved toughness of the blend. When more than 10 wt % EGMA was added to a PLA/PBS blend, the morphology of the impact surfaces changed significantly, shown in Figure 6d–f with numerous cavitation and clear plastic. In particular, it can be seen that the large number of fibers are stretched, indicating plastic deformation of 70/30/20 samples, which are able to absorb a large amount of impact energies and lead to increased toughness.

### 3.4. Compatibilizer EGMA Localization in the PLA/PBS Blend Analysis

For a multi-phase blend system, the selective localization of each phase has a great influence on the morphology, and consequently affects the mechanical properties of the blend [31]. Many researchers have conducted theoretical research on phase localization predictions. Among them, the simplest and most widely applied method is the spreading coefficient model proposed by Harkins [32]. He established a spreading coefficient mathematical equation to predict phase selective localization for ternary blend:

(1) $$\lambda_{ABC}=\gamma_{AC}-\gamma_{AB}-\gamma_{BC}$$

where λ_ABC_ is the spreading coefficient, A, B, and C represent EGMA, PLA, and PBS phases, respectively. γ_PLA/PBS_, γ_EGMA/PLA_ and γ_EGMA/PBS_ are the interfacial tensions between the two different phases, respectively, which can be calculated by the following Equations (2–6) found by Wu [33]:

(2) $$\gamma_{H_{2}O}(1+\cos\theta_{H_{2}O})=4\left(\frac{\gamma_{H_{2}O}^{d}\cdot \gamma_{{}^{}}^{d}}{\gamma_{H_{2}O}^{d}+\gamma_{{}^{}}^{d}}+\frac{\gamma_{H_{2}O}^{p}\cdot \gamma_{{}^{}}^{p}}{\gamma_{H_{2}O}^{p}+\gamma_{{}^{}}^{p}}\right)$$

(3) $$\gamma_{CH_{2}I_{2}}(1+\cos\theta_{CH_{2}I_{2}})=4\left(\frac{\gamma_{CH_{2}I_{2}}^{d}\cdot \gamma_{{}^{}}^{d}}{\gamma_{CH_{2}I_{2}}^{d}+\gamma_{{}^{}}^{d}}+\frac{\gamma_{CH_{2}I_{2}}^{p}\cdot \gamma_{{}^{}}^{p}}{\gamma_{CH_{2}I_{2}}^{p}+\gamma_{{}^{}}^{p}}\right)$$

(4) $$\gamma_{AB}=\gamma_{A}+\gamma_{B}-4\left(\frac{\gamma_{A}^{d}\cdot \gamma_{B}^{d}}{\gamma_{A}^{d}+\gamma_{B}^{d}}+\frac{\gamma_{A}^{p}\cdot \gamma_{B}^{p}}{\gamma_{A}^{p}+\gamma_{B}^{p}}\right)$$

(5) $$\gamma =\gamma^{d}+\gamma^{p}$$

(6) $$\gamma_{H_{2}O}=\gamma_{{}_{H_{2}O}}^{d}+\gamma_{{}_{H_{2}O}}^{p},\gamma_{CH_{2}I_{2}}=\gamma_{{}_{{}_{CH_{2}I_{2}}}}^{d}+\gamma_{{}_{{}_{CH_{2}I_{2}}}}^{p}$$

in which γ is the surface energy, γ^d^ and γ^p^ are the surface energy of dispersion and polar component, and θ_H_2_O_ and θ_CI_2_I_2__ are the contact angles with water and diiodomethane, respectively, PLA (θ_H_2_O_ = 66.4°, θ_CI_2_I_2__ = 42.9°), PBS (θ_H_2_O_ = 63.9°, θ_CI_2_I_2__ = 36.2°), and EGMA (θ_H_2_O_ = 109.2°, θ_CI_2_I_2__ = 61.1°). The surface energy of water and diiodomethane are γ^d^_H_2_O_ = 22.1 mN/m, γ^p^_H_2_O_ = 50.7 mN/m, γ^d^_CI_2_I_2__ = 44.1 mN/m and γ^p^_CI_2_I_2__ = 6.7 mN/m used for reference [34].

**Figure 7:**
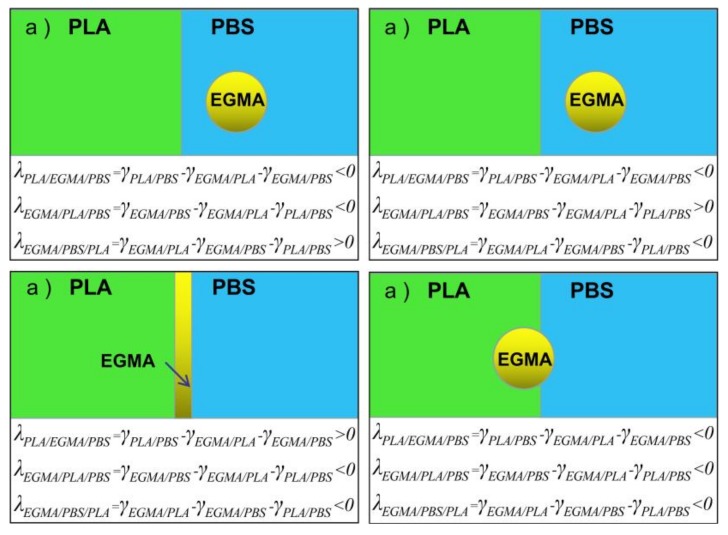
Possible morphologies predicted by spreading coefficients.

**Figure 8:**
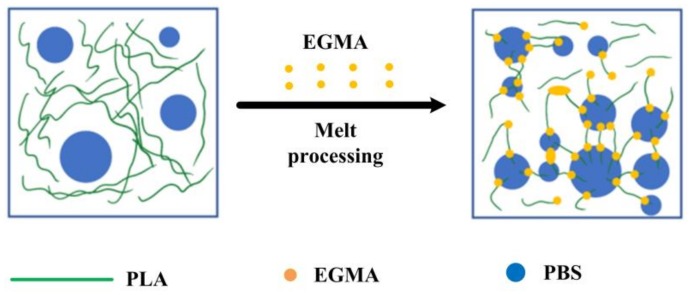
Schematic illustration of the morphology evolution PLA/PBS blend with the inclusion of EGMA.

According to the above theoretical analysis, the interfacial tensions and spreading coefficients between components can be calculated and are listed in Table 1. The γ_PLA/PBS_ is higher than γ_PBS/EGMA_ and γ_PLA/EGMA_, showing the poor compatibility between PLA and PBS, attributed to the higher the interfacial tension value and the weaker interaction between the components [31]. Furthermore, γ_PLA/EGMA_ is lower than γ_PBS/EGMA_, indicating that the compatibility between PLA and EGMA is better than PBS and EGMA. In the meantime, the spreading coefficient values of λ_PLA/EGMA/PBS_, λ_EGMA/PLA/PBS_ and λ_EGMA/PBS/PLA_ are all negative. According to the possible morphologies predicted by spreading coefficients [32] shown in Figure 7, the EGMA phase locates at the phase interface between PLA and PBS in spherical form, while some spherical particles exist in the PLA phase as presented in Figure 7d. In summary, the schematic illustration of possible morphologies predicted by the PLA/PBS blend with the inclusion of EGMA is shown in Figure 8.

**Figure 9:**
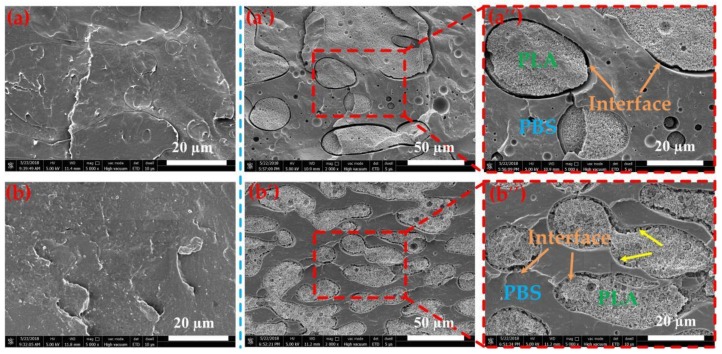
SEM images of cryofracture surfaces and PLA amorphous were etched cryofracture surfaces:(**a**–**a”**) PLA/PBS 50/50; (**b**–**b”**) PLA/PBS/EGMA 50/50/10.

Based on the above theoretical analysis, the EGMA as spherical particles is distributed at the PLA-PBS interfaces. In order to verify the correctness of theoretical analysis, studying the phase morphology of polymers is an essential proof. Due to the different mass fractions of each component in PLA/PBS/EGMA blends, it is difficult to determine the location of the EGMA as shown in Figure 5. To visually determine the location of the EGMA, 50/50 PLA/PBS and 50/50/10 PLA/PBS/EGMA blends were prepared, and the SEM images of cryofracture surfaces and PLA amorphous etchings can be found in Figure 9. In Figure 9a, the interface clearly exists on boundaries of PLA and PBS phases. Meanwhile, there are many large voids and no interconnection between PLA and PBS phases are shown in Figure 9a’–a”, since the incompatibility of PLA and PBS has been proven above. With the incorporation of 10 wt % EGMA shown in Figure 9b, the cryofracture surface exhibited a very smooth surface with the disappearance of phase interfaces. In particular, when the PLA amorphous was etched, it is clearly observed from Figure 9b’–b” that a large number of EGMA particles are distributed at the interface between PLA and PBS, connecting with both PBS and PLA crystals, thus proving that the previous theoretical analysis is correct.

### 3.5. Toughening Mechanism

It is known to all that the efficiency of the flexible material in toughening the PLA significantly relies on the interfacial compatibility between dispersed phase and PLA matrix. Poor compatibility leads to low interfacial adhesion between the two phases; when subjected to tensile or impact, phase separation easily occurs, and the toughening effect is not achieved. Nevertheless, compatibility that is too high is also not good for toughness because it does not permit stress to be alleviated with high interfacial adhesion. Therefore, in this part, the toughening mechanism of the PLA is established by investigating the morphology and interfacial compatibility of the PLA/PBS blend with the inclusion of EGMA.

The elongation at break is an important indicator for evaluating toughness. According to mechanical tests shown in Figure 3, it can be observed that the elongation at break of PLA significantly increased with the inclusion of PBS, which is due to the PBS is the soft material that can lead to a toughening effect. When EGMA is added to PLA/PBS blend, the elongation at break continues to increase significantly. In particular, with 10 wt % EGMA, the sample exhibits an elongation at break that reaches 549.4%. Such improvement is attributed to the occurrence of in situ reaction between PLA and PBS phases with the inclusion of EGMA, which can improve the compatibility between PLA and PBS. However, with further increase of EGMA loading, the elongation at break of PLA/PBS blend containing 20 wt % is only 324.1%, showing a sharp downward trend that could be ascribed to the changes in the size of dispersed phase domains. Owing to the dispersed PBS particles acting as stress concentrators in PLA/PBS and PLA/PBS/EGMA blends [35], the size and distribution of the dispersed phase particles are other important factors affecting the elongation at break besides interfacial adhesion [36].

**Figure 10:**
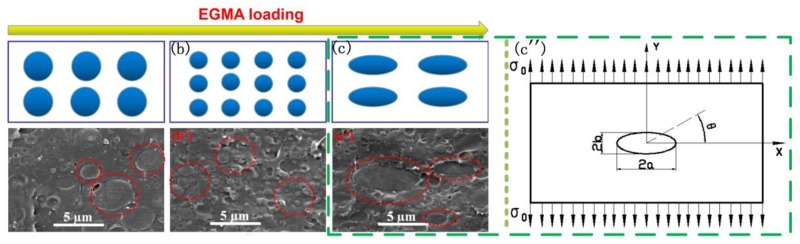
Evolution of PBS particle shape in phase morphology with the inclusion of EGMA:(**a,a’**) PLA/PBS 70/30; (**b,b’**) PLA/PBS/EGMA 70/30/10; (**c,c’**) PLA/PBS/EGMA 70/30/10; (**a**–**c**) schematic diagram; (**a’**–**c’**) SEM images of cryofracture surfaces; (**c”**) analysis model.

The evolution of PBS particle shape in phase morphology with the inclusion of EGMA is shown in Figure 10. It can be seen that the large PBS spherical particles are distributed in the PLA matrix. For samples with EGMA less than 10 wt %, the PBS particles distributed uniformly were accompanied by decreased particle size presented in Figure 10b; the dispersed PBS particles that act as stress concentrators reduced the number of stress concentrations, and led to improved elongation at break [37]. With further increase of EGMA content, the morphology changed significantly where the PBS particles increased in particle size and gradually evolved from spherical to ellipsoidal as shown in Figure 10c, and the ellipsoidal particles were unevenly distributed in the matrix act as some flaws in PLA/PBS/EGMA ternary blends caused stress concentration, which could be have an effect on the reduction in elongation at break. For the sake of investigating the relationship between the change of phase morphology and the elongation at break, the stress analysis model elliptical shape was presented in Figure 10c”; Han et al. [38] established the stress equation at the edge of the hole as follows:

(7) $$\sigma_{\theta }=\frac{1-p^{2}-2p+2\cos2\theta }{1+p^{2}-2p\cos2\theta }\sigma_{0}$$

in which, *p* = (a − b)/(a + b), a and b are the length of the major and minor axis of the ellipsoidal hole, σ_0_ is the average stress, and θ is the angle of the dot on the edge of the ellipsoidal hole to the *x*-axis. The maximum normal stress can be obtained as follows:

(8) $$\sigma_{\max}=\sigma_{\theta }{}_{=\pm \pi /2}=\frac{3-p}{1+p}\sigma_{0}=(1+\frac{2a}{b})\sigma_{0}$$

It can be seen that the maximum stress of the circular hole (a = b) is three times more than the average stress, and the larger the value of a/b, the larger σ is. Therefore, the maximum stress of the elliptical hole is larger than that of the circular hole, which shows that the stress concentration of the elliptical hole is more serious, resulting in a sharp decrease in elongation at break.

The impact strength is another important indicator for evaluating toughness. As can be seen in Figure 4—that the impact strength of PLA increased with the value of 7.3 kJ/m^2^—while the improvement is not obvious, this is due to the poor compatibility of the PBS particles in the PLA matrix as shown in the SEM image of cryofracture surface in Figure 5a. Meanwhile, it can be clearly observed that the large voids surrounding spherical PBS are surrounded by PLA crystals, with no interconnection between two phases. Furthermore, many PBS particles detached from the PLA matrix and PLA amorphous was etched in cryofracture surfaces in Figure 5a’’, which indicated that the two phases were immiscible. When the sample is subjected to the impact process, the poor interfacial adhesion leads to interface detachment, limiting the increase of toughness. However, the impact strength has been significantly improved when EGMA was added to a PLA/PBS blend. In particular, the impact strength has increased nearly 20 times compared to neat PLA with incorporation of 20% EGMA, which was due to EGMA improving the interfacial compatibility between PLA and PBS phases. The FT-IR spectra indicated that the epoxy group of EGMA reacts with the hydroxyl groups of PLA and PBS, and the EGMA distributed at the interface between PLA and PBS phase acted as a bridge to connect two phases presented in Figure 5 and Figure 9. At the same time, the specimens of each blend undergo the impact test as shown in Figure 4B–C. The neat PLA and PLA/PBS impact specimens are completely broken, while PLA/PBS blends with the added EGMA are not completely separated. In particular, the stress-whitened areas appeared around impact-fractured surfaces, and this area gradually became larger as the EGMA content increased. In addition, the impact interface also became rough gradually as presented in Figure 4Bc–f, which indicated the improvement of the two-phase compatibility, and the results are consistent with those of impact strength.

## 4. Conclusions

In the work, high toughness biodegradable PLA/PBS blends with compatibilizer EGMA were successfully fabricated by melt blending using a self-made triple screw extruder. The mechanical properties, morphology, and interfacial compatibility of the blends with various EGMA content were investigated. The FT-IR spectra results indicated that the epoxy group of EGMA reacts with the hydroxyl groups of PLA and PBS, which proved the occurrence of interfacial reactions among the tri-components. The morphology of PLA/PBS blend revealed that the two phases are immiscible. With EGMA added to the PLA/PBS blends, obvious morphology changes were observed, indicating a significantly improved compatibility and interfacial adhesion. Meanwhile, the selective prediction of spreading coefficient was obtained through a contact angle test, which indicated that the EGMA was selectively localized at the phase interface between PLA and PBS phase in the form of spherical particles, which was verified by morphology of cryofracture and PLA amorphous being etched into cryofracture of surfaces PLA/PBS (sample 50/50) and PLA/PBS/EGMA blend (sample 50/50/10). Finally, the excellent compatibility leads to a significant increase in the toughness of PLA ternary polymer. Based on the mechanical properties’ test results, the elongation at break and impact strength of PLA/PBS blends reached the maximal value of 549.4% and 46.5 kJ/m^2^ when 10 wt % and 20 wt % EGMA were added, exhibiting superior stretchability of 83 times and 20 times higher than neat PLA, respectively. Ultimately, the toughening mechanism of PLA based polymers was established based on the above analysis, exploring a new way for the extensive applications for degradable material.

## Figures and Tables

**Table 1:** The prediction parameters of phase selective localization of PLA, PBS and EGMA.

Sample	Surface Tension (mN/m)			Interfacial Tension (mN/m)	Spreading Coefficient (mN/m)
	Total (λ)	Dispersion (λ_d_) (cd)	Polar (λ_p_)		
**PLA**	42.29	33.27	9.02	γ_PLA/PBS_ = 0.9	λ_PLA/EGMA/PBS_ = −9.36
**PBS**	45.46	36.44	9.01	γ_PBS/EGMA_ = 5.24	λ_EGMA/PLA/PBS_ = −1.12
**EGMA**	30.72	30.11	0.61	γ_PLA/EGMA_ = 5.02	λ_EGMA/PBS/PLA_ = −0.68
